# Supplementary material for: Multi-level, forming and filament free, bulk switching trilayer RRAM for neuromorphic computing at the edge
Source: Nat Commun. 2024 Apr 25;15:3492. doi: 10.1038/s41467-024-46682-1 (PMC11045755; doi:10.1038/s41467-024-46682-1)
Supplement: Supplementary file 1 — Supplementary Information [file 41467_2024_46682_MOESM1_ESM.pdf]

Supplementary Figures for "Multi-level, Forming and Filament Free, Bulk Switching Trilayer RRAM for Neuromorphic Computing at the Edge"

Jaeseoung Park[1], Ashwani Kumar[1], Yucheng Zhou[1], Sangheon Oh[1], Jeong-Hoon Kim[1], Yuhan Shi[1], Soumil Jain[2], Gopabandhu Hota[1], Erbin Qiu[3], Amelie L. Nagle[4], Ivan K. Schuller[3], Catherine D. Schuman[4], Gert Cauwenberghs[2] and Duygu Kuzum[1]*

[1]Department of Electrical and Computer Engineering, [2]Department of Bioengineering, [3]Department of Physics, University of California, San Diego, CA, USA. [4]Department of Computer Science, Massachusetts Institute of Technology, MA, USA.

[5]Department of Electrical Engineering and Computer Science, University of Tennessee, TN, USA.

The authors contributed equally: Jaeseoung Park, Ashwani Kumar.

* Corresponding Author: Duygu Kuzum, email: dkuzum@ucsd.edu

Supplementary note 1

Our bulk switching RRAM consists of a tunnel barrier Al_2O_3 layer and a space charge limited conduction TiO_x layer. They are connected in series, so they follow the current and voltage equations below.

$$J_{tot} = J_{Al2O3} = J_{TiOx}, V_{tot} = V_{Al2O3} + V_{TiOx} \quad \text{(S1)}$$

Through the Al_2O_3 tunnel barrier, direct tunneling or FN tunneling can occur depending on the voltage applied and electric field across the Al_2O_3.[1,2] The current conduction through the TiO_x SCLC layer occurs through carrier drift and SCLC with different power law dependencies on voltage Ohm's law (J∝V) and Mark-Helfrich's law ($J \propto V^{m+1}$), respectively.[3-5] The equations for all these mechanisms are as follows.:

$$J_{Al2O3} = J_{DT} + J_{FN} \quad \text{(S2)}$$

$$J_{DT} = \left(\frac{e^2}{sh^2}\right)[m^*(\varphi_1 + \varphi_2)]^{\frac{1}{2}} V \times \exp\left(-\left(\frac{4\pi s}{h}\right) m^{*\frac{1}{2}} (\varphi_1 + \varphi_2)^{\frac{1}{2}}\right) \quad \text{(S3)}$$

$$J_{FN} = \left(\frac{e^3V^2}{8\pi h\Phi}\right)\exp\left[\frac{-8\pi(2m^*)^{\frac{1}{2}}\Phi^{\frac{3}{2}}}{3heV}\right] \quad \text{(S4)}$$

$$J_{TiOx} = J_{Drift} + J_{SCLC} \quad \text{(S5)}$$

$$J_{Drift} = en\mu V, n \propto \exp\left(-\frac{1}{kT}\right) \quad \text{(S6)}$$

$$J_{SCLC} = Ne\mu\left(\frac{\varepsilon_s\varepsilon_0}{eN_t}\right)^m \left(\frac{m}{m+1}\right)^m \left(\frac{2m+1}{m+1}\right)^{m+1} \frac{V^{m+1}}{d^{2m+1}}, m = T_c/T \quad \text{(S7)}$$

Where e is the electron charge, s is the Al_2O_3 layer thickness, h is the Planck constant, $m^*$is electron effective mass, φ_1 and φ_2 are barrier heights of Al_2O_3 layer from metal and TiO_x sides respectively, Φ is average barrier height of Al_2O_3 layer, n is the free electron density, μ is the mobility of electron, N_c is the effective density of state in the conduction band, ε_s is the dielectric constant of TiO_x, ε_0 is the permittivity of vacuum, N_t is the trap density, d is the TiO_x layer thickness, T_c is the characteristic temperature, m is T_c/T. T_c is the characteristic temperature defining the slope of the exponential trap distribution over bandgap energy.[3]

Supplementary note 2

To quantitatively analyze the device non-linearity, we investigated non-linearity of the trilayer bulk RRAM devices using the following equations.[6]

$$G_{LTP} = B\left(1 - e^{\left(-\frac{P}{A}\right)}\right) + G_{min}, G_{LTD} = -B\left(1 - e^{\left(\frac{P-P_{max}}{A}\right)}\right) + G_{max} \quad \text{(S8)}$$

$$B = (G_{max} - G_{min})/(1 - e^{\frac{-P_{max}}{A}}) \quad \text{(S9)}$$

We demonstrated weight updates using both identical and incremental pulse schemes. When we adopt the identical pulse scheme, the non-linearity values of +3.68 and -4.34 (Fig. S3a) during the potentiation and depression were achieved. The non-linearity could be improved to -1.24 and -4.21 (Fig. S3b) for the potentiation and depression process by using the incremental pulse scheme. We expect that the non-linearity could be further improved by optimizing the pulse amplitude and width for potentiation and depression in the incremental scheme. Furthermore, there are algorithm-device co-design approaches that can be adopted to compensate system-level effects of switching nonlinearity. For instance, to mitigate the non-linearity effect on accuracy drop in online learning and classification tasks, we previously developed the adaptive quantization method, which maps weights onto the device conductances based on the distribution and relative-importance of the weights.[7] Various other nonuniform quantization methods have also been adopted by the broader neural networks community to improve efficiency of neural networks.[7]

Supplementary Note 3

The current conduction occurs through direct tunneling, FN tunneling, and SCLC models. Based on these models, we simulated the current density under the electric field with various tunneling barrier (Al_2O_3) thickness layers. We varied tunneling barrier thickness from 20 Å to 40 Å, and the current density of them were plotted in Fig. S8a. As the current density in the direct tunneling exponentially decays with the oxide thickness, we expect the current density to be decreased by around 3 orders of magnitude per 1 nm Al_2O_3 thickness. These simulation results suggest that to set the device resistance to in ~MΩ regime, the Al_2O_3 tunneling barrier thickness should be chosen ~30Å. So, we have decided 30Å of Al_2O_3 tunneling barrier to make our devices in ~MΩ regime. We plotted the current density of an experimentally measured RRAM device with 30Å tunneling barrier thickness and 5μm diameter (shown with open circle in Fig. S8a) showing 1MΩ resistance at 100 mV read voltage, showing great consistency with predictions based on the tunneling current calculations. Our methodology suggests that there is more room to modulate the barrier thickness depending on the target resistance and the device size.

Once the tunnel barrier thickness is fixed, we systematically optimized the thickness of SCLC conduction layer made of TiO_x. Thinner TiO_x will result in higher electric field across the SCLC layer for the same applied voltage and hence will increase the chances clustering of oxygen vacancies through drift to form filaments. To observe this, we chose two different TiO_x thicknesses (6.5 nm for S3 and 40 nm for S4) leading to a difference in the applied electric field across the SCLC layer. We fitted the J-V data of both S3 and S4 using tunneling and SCLC conduction models, and we extracted the electric field applied across the TiO_x switching layer shown in Fig. S8b below. Since S3 has a thinner sputtered layer than S4, it causes a higher electric field across the TiO_x switching layer. The electroforming step in filamentary RRAM devices corresponds to the controlled soft-breakdown process in a thin insulator film.[8,9] Therefore, the thin oxide film or high electric field are known to induce facile filament formation behavior in the RRAM devices. Due to high electric field in S3, it causes filament forming under a high voltage regime ($|V| > 1V$) as seen in Fig. 1g in the manuscript because the V_O filaments protrude the whole switching layer. This

filamentary switching mechanism is the same as the ALD bilayer filamentary RRAM devices (S1 and S2). In a lower voltage regime (|V| < 1V) where electric filed is not large enough to drift and cluster V_O, it shows bulk switching behavior through modulation of V_O distribution across the switching layer. In S4, however, we successfully suppressed the filament formation by reducing the electric field with a thick and amorphous TiO_x layer so that we could achieve stable bulk switching behavior.

The difference between S3 and S4 is the applied electric field across the sputtered TiO_x layer. We fitted the J-V data of both S3 and S4 using tunneling and SCLC conduction models, and we extracted the electric field applied across the TiO_x switching layer (Fig. S8b). Since S3 has a thinner sputtered layer than S4, it causes a higher electric field across the TiO_x switching layer. The electroforming step in filamentary RRAM devices corresponds to the controlled soft-breakdown process in a thin insulator film. Therefore, the thin oxide film or high electric field are known to induce facile filament formation behavior in the RRAM devices. Due to high electric field in S3, it causes filament forming under a high voltage regime (|V| > 1V) because the V_O filaments protrude the whole switching layer. This filament switching mechanism is the same as the ALD bilayer filamentary RRAM devices (S1 and S2). In a lower voltage regime (|V| < 1V), it shows bulk switching behavior due to a change in defect distribution across the switching layer. In S4, however, we successfully suppressed the filament formation with a thick and amorphous TiO_x layer so that we could achieve stable bulk switching behavior.

Supplementary note 4

Left three columns of the table compares relatively mature filamentary RRAM technologies that consist of widely used RRAM materials HfO_x, TaO_x, AlO_x, or TiO_x.[10-12] Filamentary RRAM devices need an initial high voltage forming step (V > 2.0V) to generate a conductive filament that is not compatible with advanced CMOS technology nodes. When the filaments are formed through the switching layer, the device switches to a low resistance state (LRS) and shows a few KΩ of LRS resistance (R_{ON}). The low R_{ON} not only constrains the array size and number of parallel MAC operations but also increases the read energy consumption and limits the voltage drop across the selected causing unsuccessful write operations as shown in Fig. S5b. Furthermore, filamentary RRAM devices need complex read and verify schemes to program the devices to the target conductance states which substantially increases the overall energy consumption and limits the online learning capability using these devices. In addition, filamentary RRAM devices can achieve a small number of states, providing precision less than 4-bit which is not sufficient for most neuromorphic computing applications.

Right three columns compare recently developed bulk RRAM devices based on $NdNiO_3$, $Pr_{0.7}Ca_{0.3}MnO_3$ and $Al_2O_3/TiO_2/TiO_x$ to address the limitations of filamentary RRAM devices explained above.[13,14] First, these bulk RRAM technologies do not need any initial high voltage forming step. The resistance modulation occurs in MΩ regime ensuring stable and reliable large size array operations. The cell size of these bulk RRAM is larger than the filamentary RRAM since the technology is less mature and has not yet translated into foundries in contrast to HfO_x based filamentary RRAM. Since all bulk RRAM devices exhibit higher resistance than filamentary RRAM, the energy consumption is much lower for the read operation. In addition, many number of states could be achieved to represent the synaptic weights in neuromorphic computing applications. Among the three bulk RRAM technologies, only our work presents a large array-scale demonstration. Furthermore, perovskite-based bulk RRAM technologies need high process temperature (T > 500°C) so further process development to enable fabrication at low temperatures (T < 400°C) will be necessary for CMOS BEOL compatibility. Our bulk RRAM devices based on

Al_2O_3/TiO_2 materials not only address the limitations of the mature filament RRAM technologies but are also compatible with integration at the CMOS BEOL to enable a high-density 3D compute-in-memory platform for neuromorphic applications. Based on Table S2, the key figures of merit of our bulk RRAM technology RRAM technology can be summarized as follows; forming-free operation, CMOS BEOL compatibility, high R_{on} and R_{off} that enables reliable read and write in large scale crossbar arrays and low energy operation, low switching voltages, high number of conductance states, endurance comparable to RRAM technologies, and much lower total read energy.

Supplementary Note 5

The difference between the expected MVM and the measured MVM values arises from non-ideal factors, including potential sneak paths from neighboring columns, I-R (Interconnect Resistance) drop along the BLs and WLs. Also, the switches used for driving the WLs and BLs have non-zero resistances which further contributes to the non-linearity on the output. This might induce errors when we extract the individual conductance. Moreover, the inactivated devices in the column or BL contribute to the sampled capacitor voltage during the single device conductance measurement. Such contributions from the inactive devices might be reflected in the extracted single device conductance, G_{target}, in equation (3) in method, which further introduces calculation errors in the expected MVM value. Thus, all these factors contribute to the deviation between expected and measured MVM.

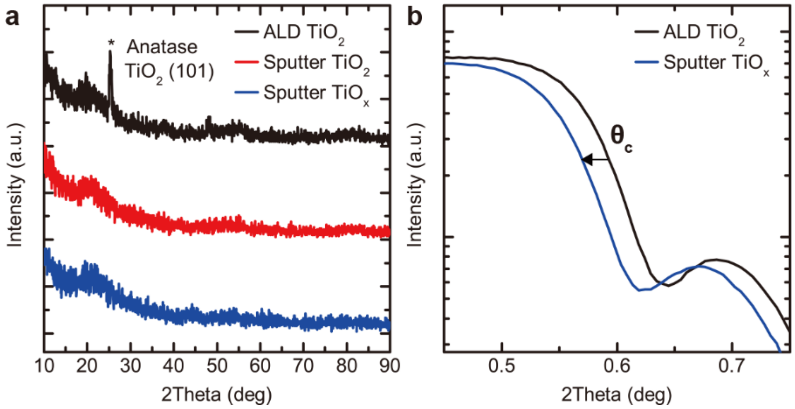

Figure S1. TiO_2 characterization using X-ray diffraction. **a**. Grazing-Incidence XRD scans of 30 nm-thick ALD and sputtered TiO_2 films. ALD TiO_2 shows a crystalline anatase phase and sputtered TiO_x films show an amorphous phase. **b**. X-ray reflection (XRR) measurements of different TiO_x films. Sputter TiO_x film has lower film density than ALD TiO_2 film.

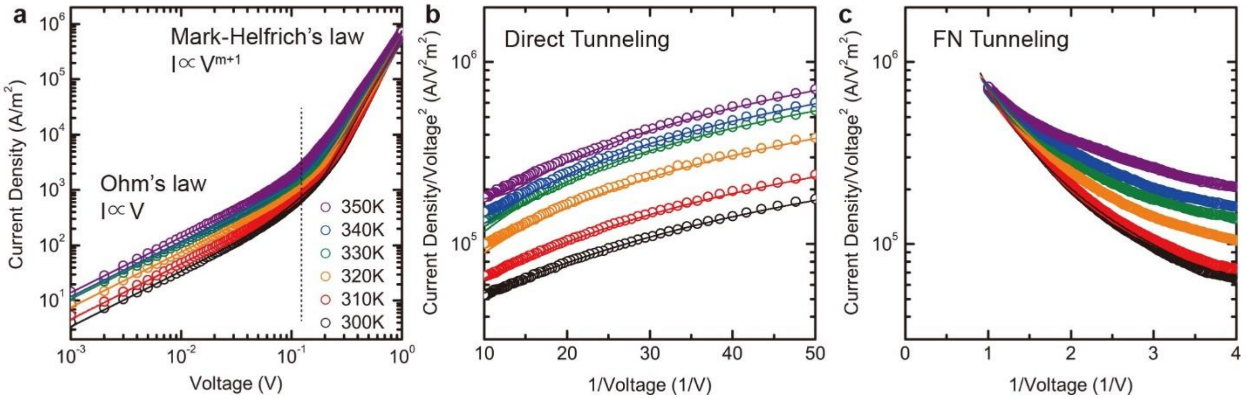

Figure S2. Experimentally measured I-V and fitted curve with different temperature from 300K to 350K. **a**. Whole range, log J – log V curves. **b**. Magnified image of log(J/V^2) vs. 1/V curves from 0V to 0.1V. **c**. Magnified image of log(J/V^2) vs. 1/V curves from 0.25V to 1V. Experimentally measured data (dotted circle) are fitted with our electrical conduction model (line).

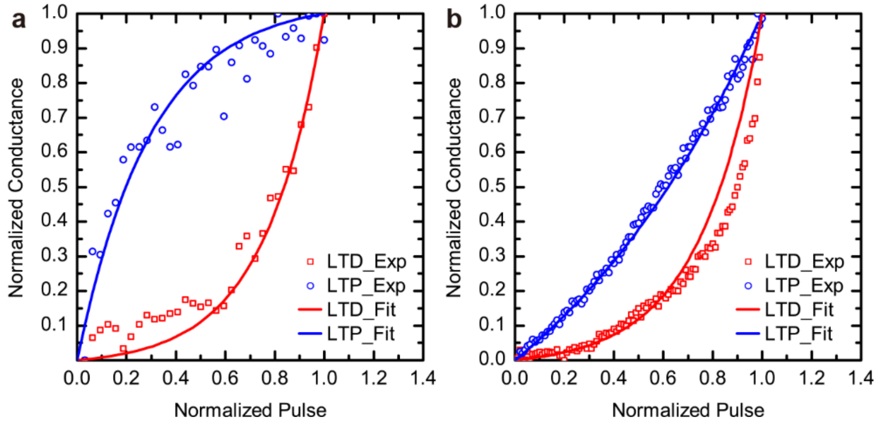

Figure S3. Normalized conductance vs. Normalized pulse using **a**. identical and **b**. incremental pulse schemes.

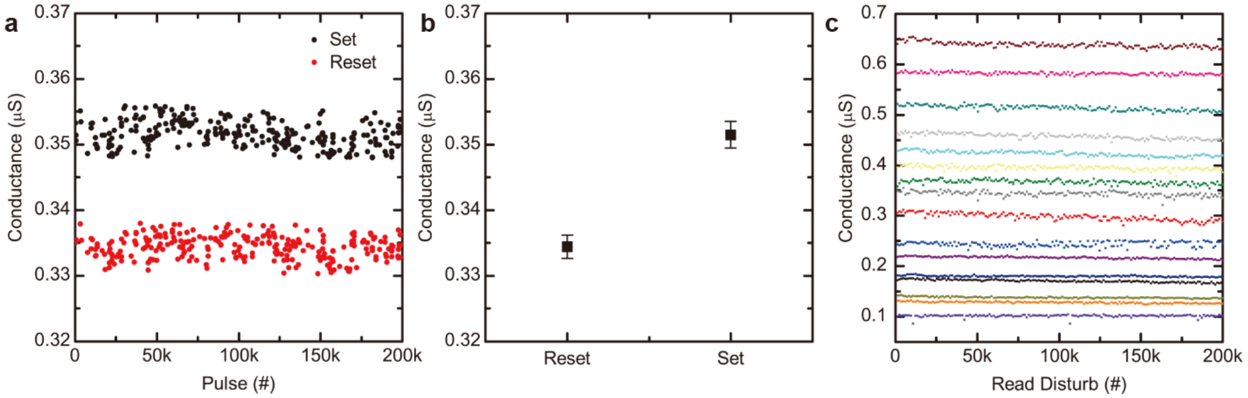

Figure S4. **a**. Endurance and **b**. read noise during the endurance tests up to 2×10^5 pulses. **c**. read disturb over 200k cycles for trilayer RRAM.

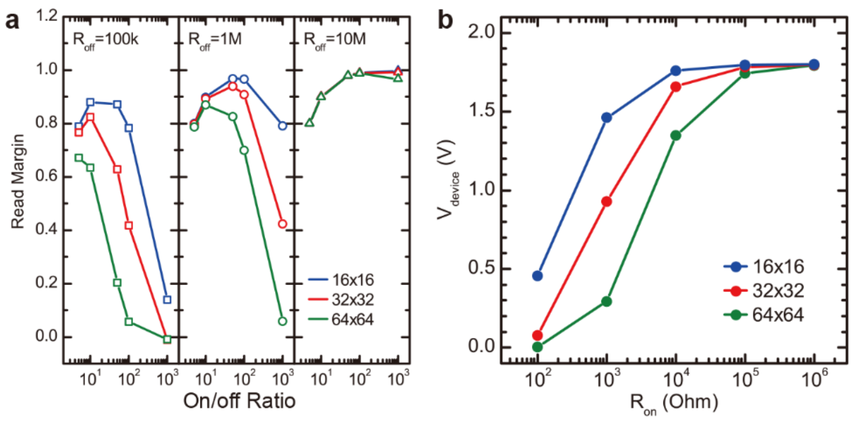

Figure S5. **a**. Read margin for different R_{OFF} and array size simulated for the worst-case (selected cell is the farthest from the word-line/bit-line voltage sources) using $V_{dd}/2$ scheme. **b**. Voltage falling across device (V_{device}) decreases with R_{ON}, especially for large arrays. 1.8V applied to WL and switching threshold was assumed 1.5 V.

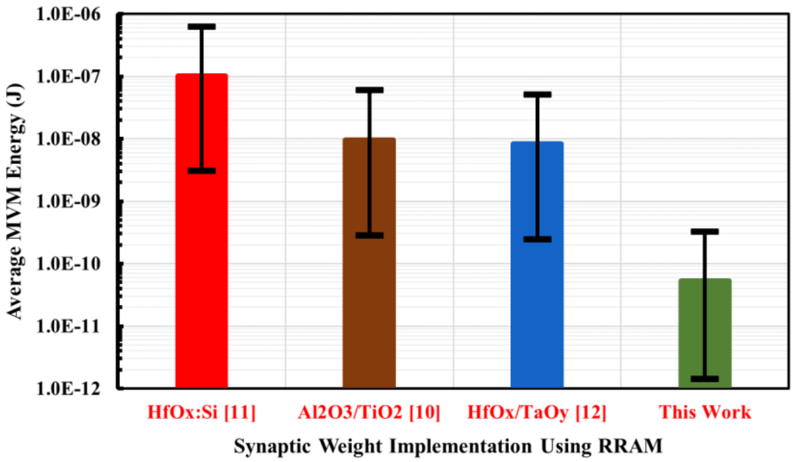

Figure S6. Average energy consumption and its variance during navigation of all 15 racetracks by using different RRAM technologies for implementing synaptic weights.

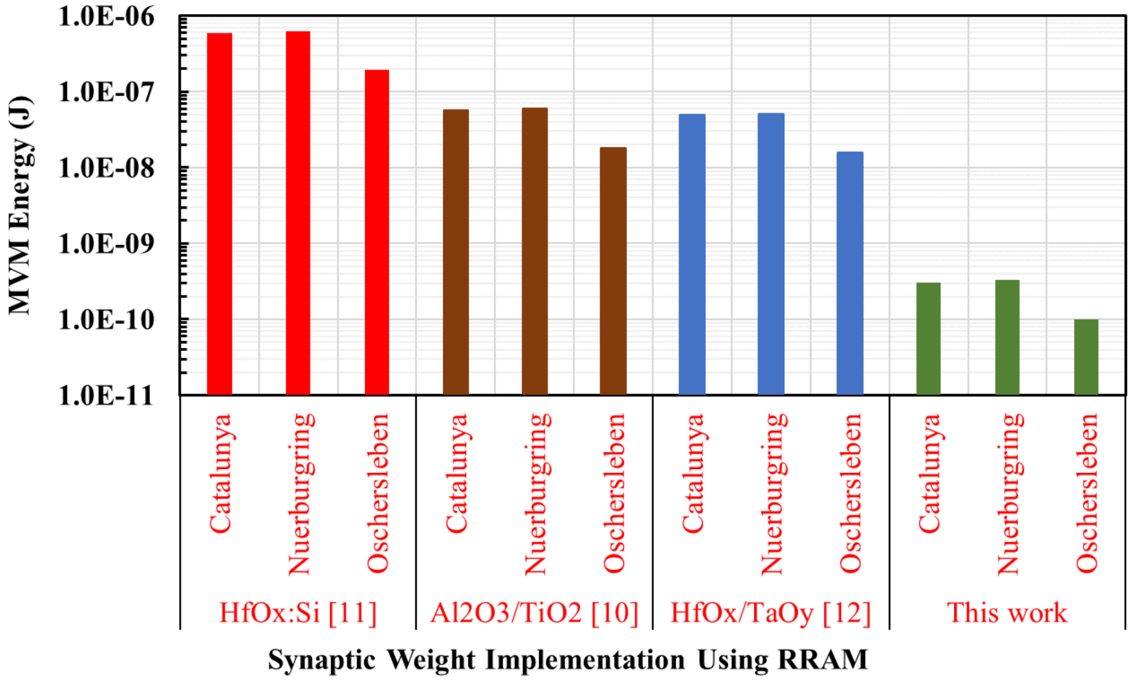

Figure S7. Total energy consumption during navigation of three representative racetracks by using different RRAM technologies for implementing synaptic weights.

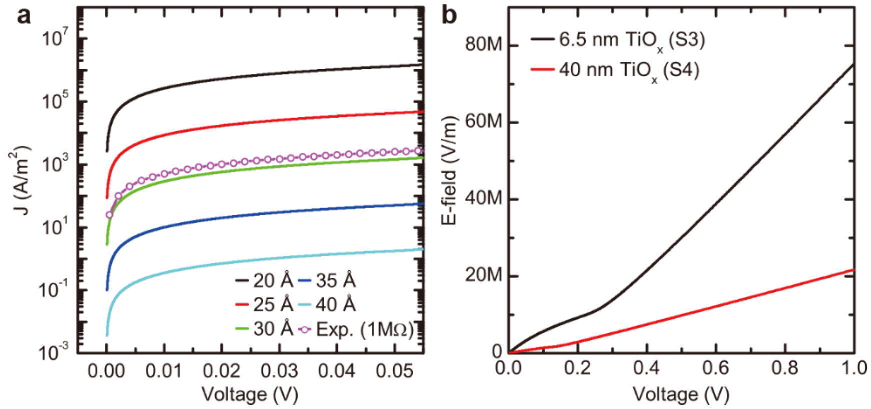

Figure S8. **a**. Current density versus applied voltage across the RRAM switching layer. The Al_2O_3 thickness was varied from 20Å to 40Å to determine the experimental Al_2O_3 thickness. Open circle curve shows experimentally measured current density from an RRAM device with 30Å tunneling barrier thickness and 5µm diameter, exhibiting 1MΩ resistance at 100mV read voltage. **b**. Electric field applied across the TiO_x sputtered layer in S3 and S4.

	Sheet Resistance (Ω/□)	Conductivity $(\Omega \cdot m)^{-1}$	Estimation by Rs	Estimation by O_2/Ar ratio
ALD TiO_2	125M	0.16	$TiO_{1.96}$	N/A
Sputtered TiO_x	1.48M	13.5	$TiO_{1.85}$	$TiO_{1.87\pm0.02}$

Table S1. Stoichiometry estimation by conductivity measurement and XPS analysis.

We have estimated the stoichiometry of the sputtered TiO_x and ALD TiO_2 film in Table S1. We expect the stoichiometry of the sputtered TiO_x layer to be around $TiO_{1.85}$ based on our experimentally measure conductivity and O_2/Ar ratio during the sputtering process[15,16]. Due to the intentionally induced V_O defects, SCLC conduction occurs through the layer in comparatively higher electric field than where the ohmic conduction occurs in the TiO_x layer.

RRAM Technologies	Al_2O_3/TiO_2 [10]	HfO_x/TaO_y [12]	HfO_2:Si [11]	PCMO [14]	$NdNiO_3$ [13]	$Al_2O_3/TiO_2/TiO_x$ [This work]
**Switching Type**	Filamentary	Filamentary	Filamentary	Bulk	Bulk	Bulk
**Forming voltage**	~3.3V	~4.0V	~2.0V	None	None	None
**CMOS Compatibility**	✓	✓	✓	X	X	✓
**Ron**	16 KΩ	23.8 KΩ	6 KΩ	6.25 MΩ	2 KΩ	2.78 MΩ
**Roff**	333 KΩ	500 KΩ	600 KΩ	157 MΩ	4.5 KΩ	6.67 MΩ
**Cell area (μm^2)**	0.0625 μm^2	0.360 μm^2	0.04 μm^2	0.0225 μm^2	70700 μm^2	19.6 μm^2
**Switching voltage**	1.2 V/ -1.4 V	+1.6 V / -1.9 V	+1.7 V / -2.0 V	-3.0V / +3.0V	-3.0 V / 5.4 V	-1.8V / + 0.65V
**Pulse width**	1ms	10us	100ns	1ms	960ns	5ms
**# of states**	16	8	2	50	32	100
**Endurance**	10^6	10^6	10^5	10^4	-	$2*10^5$
**Total Energy per read operations**	51.0 fJ	44.02 fJ	167.7 fJ	1.04 fJ	722.2 fJ	~ 0.51 fJ

Table S2. Benchmarking of conventional RRAM technologies for neuromorphic applications.

	Sheet Resistance (Ω/□)
Center	1.48M
Middle	1.53M
Edge	1.53M

Table S3. Sheet resistance uniformity of sputtered TiO_x layer across the wafer.

To investigate uniformity of the films, we deposited 50-nm thick of sputtered TiO_x layer on a 4-inch SiO_2/Si wafer and measured the sheet resistance across the wafer. We confirmed highly uniform sheet resistance and device switching behavior across the whole wafer which can address the reproducibility and variability issues in filament RRAM devices.

References

1 Lenzlinger, M. & Snow, E. Fowler-Nordheim tunneling into thermally grown SiO2. *Journal of Applied physics* **40**, 278-283 (1969).

2 Simmons, J. G. Electric tunnel effect between dissimilar electrodes separated by a thin insulating film. *Journal of applied physics* **34**, 2581-2590 (1963).

3 Mark, P. & Helfrich, W. Space-charge-limited currents in organic crystals. *Journal of Applied Physics* **33**, 205-215 (1962).

4 Lampert, M. A. Simplified theory of space-charge-limited currents in an insulator with traps. *Physical Review* **103**, 1648 (1956).

5 Lampert, M. A. & Schilling, R. B. in *Semiconductors and semimetals* Vol. 6 1-96 (Elsevier, 1970).

6 Yu, S. Neuro-inspired computing with emerging nonvolatile memorys. *Proceedings of the IEEE* **106**, 260-285 (2018).

7 Shi, Y. *et al.* Adaptive quantization as a device-algorithm co-design approach to improve the performance of in-memory unsupervised learning with SNNs. *IEEE Transactions on Electron Devices* **66**, 1722-1728 (2019).

8 Park, T. H. *et al.* Thickness-dependent electroforming behavior of ultra-thin Ta2O5 resistance switching layer. *physica status solidi (RRL)–Rapid Research Letters* **9**, 362-365 (2015).

9 Hu, R. *et al.* Investigation of Resistive Switching Mechanisms in Ti/TiOx/Pd-Based RRAM Devices. *Advanced Electronic Materials* **8**, 2100827 (2022).

10 Kim, H., Mahmoodi, M., Nili, H. & Strukov, D. B. 4K-memristor analog-grade passive crossbar circuit. *Nature communications* **12**, 5198 (2021).

11 Grenouillet, L. *et al.* in *2021 IEEE International Memory Workshop (IMW).* 1-4 (IEEE).

12 Wan, W. *et al.* A compute-in-memory chip based on resistive random-access memory. *Nature* **608**, 504-512 (2022).

13 Zhang, H.-T. *et al.* Perovskite neural trees. *Nature communications* **11**, 2245 (2020).

14 Moon, K. *et al.* in *2015 IEEE International Electron Devices Meeting (IEDM).* 17.16. 11-17.16. 14 (IEEE).

15 El Mesoudy, A. *et al.* Band gap narrowing induced by oxygen vacancies in reactively sputtered TiO2 thin films. *Thin Solid Films* **769**, 139737 (2023).

16 Hoskins, B. D. & Strukov, D. B. Maximizing stoichiometry control in reactive sputter deposition of TiO2. *Journal of Vacuum Science & Technology A* **35** (2017).

1
